# Impact of GC content on gene expression pattern in chicken

**DOI:** 10.1186/1297-9686-45-9

**Published:** 2013-04-04

**Authors:** You Sheng Rao, Xue Wen Chai, Zhang Feng Wang, Qing Hua Nie, Xi Quan Zhang

**Affiliations:** 1 Department of Biological Technology, Jiangxi Educational Institute, Jiangxi, Nanchang 330029, China; 2 Guangdong Provincial Key Laboratory of Agro-animal Genomics and Molecular Breeding, and Key Lab of Chicken Genetics, Breeding and Reproduction, Ministry of Agriculture, South China Agricultural University, Guangzhou, Guangdong 510642, China

## Abstract

**Background:**

GC content varies greatly between different genomic regions in many eukaryotes. In order to determine whether this organization named isochore organization influences gene expression patterns, the relationship between GC content and gene expression has been investigated in man and mouse. However, to date, this question is still a matter for debate. Among the avian species, chicken (*Gallus gallus*) is the best studied representative with a complete genome sequence. The distinctive features and organization of its sequence make it a good model to explore important issues in genome structure and evolution.

**Methods:**

Only nuclear genes with complete information on protein-coding sequence with no evidence of multiple-splicing forms were included in this study. Chicken protein coding sequences, complete mRNA sequences (or full length cDNA sequences), and 5^′^ untranslated region sequences (5^′^ UTR) were downloaded from Ensembl and chicken expression data originated from a previous work. Three indices i.e. expression level, expression breadth and maximum expression level were used to measure the expression pattern of a given gene. CpG islands were identified using hgTables of the UCSC Genome Browser. Correlation analysis between variables was performed by SAS Proprietary Software Release 8.1.

**Results:**

In chicken, the GC content of 5^′^ UTR is significantly and positively correlated with expression level, expression breadth, and maximum expression level, whereas that of coding sequences and introns and at the third coding position are negatively correlated with expression level and expression breadth, and not correlated with maximum expression level. These significant trends are independent of recombination rate, chromosome size and gene density. Furthermore, multiple linear regression analysis indicated that GC content in genes could explain approximately 10% of the variation in gene expression.

**Conclusions:**

GC content is significantly associated with gene expression pattern and could be one of the important regulation factors in the chicken genome.

## Background

GC content varies greatly between different genomic regions in many eukaryotes. A striking characteristic of vertebrate genomes, such as mammals and birds, is that their GC content varies over hundreds of kilobases to megabases constituting the so-called “isochore structure” [1-3]. It is generally recognized that this highly heterogeneous GC content distribution is a fundamental level of genome organization. Many studies have demonstrated that GC content is associated with various genomic features. For example, gene density, recombination rate, distribution of repetitive elements i.e. short interspersed nuclear elements (SINE) such as Alu repeats in primates, long interspersed nuclear elements (LINE) and other transposons and retrotransposons are significantly associated with GC content [4-6].

In order to investigate whether the isochore structure of a genome influences gene expression patterns, several studies have analyzed the relationship between GC content and gene expression in man and mouse. Lercher et al. [7] reported that gene expression breadth and GC content were strongly correlated and suggested that the concentration of housekeeping genes in GC-rich isochores might be the result of selective pressure. In a study on the human genome, Vinogradov [8] showed that a correlation existed between the GC content in genes and the maximum level of gene expression among tissues but that the promoter CpG island and gene CpG levels were more strongly related to expression breadth, which suggests that the variations of GC content and CpG level could determine the characteristics of gene expression in a synergistic interplay with transcription-factor-binding sites. The positive correlation between gene expression level and GC content of coding DNA sequences (CDS), 5^′^ UTR and 3^′^ UTR sequences and at the third positions in codons (GC_3_) has also been reported in mouse [9]. However, Gonçalves et al. [10] argued that, in mammalian genomes, the CDS of ubiquitously expressed genes generally have a low GC content and are often associated with retropseudogenes. Based on different measures of gene expression (EST, SAGE and microarray) and GC content in human and mouse genomes, Sémon et al. [11] showed that the correlations (either positive or negative) between GC content and gene expression were very weak. Although these correlations were statistically significant, the authors indicated that they should be further validated and interpreted with caution. However, more recently Arhondakis et al. [12,13] reported a relatively high correlation between GC content and gene expression in man and mouse. To date, this question is still a matter for debate.

The origins of mammals and birds can be traced back to a common ancestral species, 300–350 million years ago [14]. Among the avian species, chicken (*Gallus gallus*) is the best studied representative with a complete genome sequence. The distinctive features and organization of its sequence make it a good model to explore important issues in genome structure and evolution. Our aim is to investigate whether the highly heterogeneous distribution of GC content along the chicken genome does significantly influence the pattern of gene expression. To date, no systematic examination of the impact of GC content on the pattern of gene expression in chicken has been performed. In this study, we analyzed the correlation between GC content and gene expression for the whole chicken genome. Our data demonstrate that the GC content of 5^′^ UTR is positively and significantly correlated with gene expression level, gene expression breadth, and maximum gene expression level, whereas the GC contents of CDS, introns and GC_3_ are negatively correlated with gene expression level and breadth.

## Methods

### Sequence data

In this study, only nuclear genes with complete information on protein-coding sequence and with no evidence of multiple-splicing forms were included. Gene sequences were downloaded from the NCBI RefSeq database ( ftp://ftp.ncbi.nlm.nih.gov/genomes/Gallus_gallus/). Coding DNA sequences (CDS), complete mRNA sequences (or full length cDNA sequences), and 5^′^ UTR sequences corresponding to all annotated genes in the chicken genome were downloaded from Ensembl. For genes with a single splicing isoform, CDS length should be equal to the total length of all exons. Thus, we discarded all sequences showing a length difference of at least one base. Genes with a CDS that did not begin with an ATG start codon, or did not have a length > 300 bp, or did not occur in multiples of three nucleotides, or contained an internal stop codon, were also discarded. The 5^′^ UTR of a gene is often the least well-defined region. In this 5^′^ UTR data collection, only genes with known protein products (rather than with novel or predicted transcripts) were included, which ensured that the 5^′^ and 3^′^ ends of the analyzed 5^′^ UTR sequences were experimentally supported. Another potential risk is that 5^′^ UTR sequences may be subject to annotation errors. In order to circumvent this problem, we retrieved the full length of cDNA sequences (or the complete mRNA sequences) corresponding to the above correctly annotated genes, then aligned the complete CDS to cDNA sequences by BLAT. 5^′^ UTR that were not consistent with the alignment results were excluded. For each gene, the GC contents of the CDS, introns, and 5^′^ UTR were determined. The GC_3_ were calculated by codonW 1.4.2.

### Expression data

Chicken expression data taken from a previous study [15] included data for 19 tissues i.e. blood, brain, bursa of fabricius, cecum, connective tissue, embryonic tissue, epiphyseal growth plate, gonad, head, heart, limb, liver, muscle, ovary, pancreas, spleen, testis, and thymus. Three indices i.e. expression level, expression breadth and maximum expression level were used to measure the expression pattern of a given gene. For a given gene, expression level is the number of EST counts in all tissues, expression breadth is the number of tissues in which EST are found and maximum expression level is the highest expression value observed among the 19 tissues.

### Identification of CpG islands

CpG islands were identified using hgTables of the UCSC Genome Browser ( http://genome.ucsc.edu/). The following criteria were used: GC content ≥ 55%, Obs_CpG_/Exp_CpG_ ≥ 0.65, and length ≥ 500 bp [16,17]. Our analysis focused on the CpG islands of the 5^′^ regions of the genes selected, and more specifically of the promoter regions. Although giving a precise definition of the promoter region of a gene is an unsolved issue, in most cases it is a short region (e.g., 200 bp) located immediately upstream of the transcription start site (TSS) [18,19]. Furthermore, since in eukaryotes the average length of 5^′^ UTR is about 100–200 bp [5,20], we retrieved a 2 kb long sequence immediately upstream of the first coding site for each gene and if it contained a CpG island, this gene was regarded as CpG island-positive.

### Recombination rate estimate

The recombination rates for 1 Mb windows were estimated using the NCBI build 2.1 (released November, 2006) of the chicken genome assembly and the latest chicken consensus linkage map (sex-averaged map) [21]. The location of individual markers was determined based on the alignment of the full marker sequence using BLAST. For each 1 Mb sliding window, a linear function was fit to the points representing genetic and physical map positions and the slope of this line was interpreted as an estimation of the recombination rate. For further details see [15].

### Statistical analysis

Correlation analysis between variables was performed by SAS Proprietary Software Release 8.1. In order to assess the actual strength of associations, correlation coefficients reported in this study were obtained using all genes independently, instead of subdividing genes into groups and then investigating the relationships among them. The significance tests were corrected for multiple testing by the Bonferroni step-down correction [22]. To determine the variables contributing to expression patterns and how they may interact, we performed multiple linear regressions with the variables, excluding those not contributing significantly through the use of the t-statistical logarithm with backward stepwise regression.

The experimental protocols were approved by the Animal Care and Protection Committee of South China Agricultural University.

## Results

In order to accurately estimate the GC content of gene compartments, only nuclear genes with complete information on protein-coding sequence and with no evidence of multiple-splicing forms were included. The sequence collection contained 8631 CDS, each corresponding to a unique gene in the *Gallus gallus* genome. The 5^′^ UTR sequences dataset included only the genes with known protein products (see Methods). A recent study in man and mouse demonstrated that the 5^′^ UTR of approximately 50% of genes contain an upstream open reading frame (uORF), suggesting that uORF are phylogenetically widespread regulatory elements in the 5^′^ UTR of eukaryotic genes [23]. Since a considerable fraction of the 5^′^ UTR contain an uORF at least 10 codons long [24], we considered only entries with complete 5^′^ UTR sequences spanning from the cap site to the start codon (excluded) with a length ≥ 30 bp representing 3575 genes.

We calculated the GC content of the CDS, introns, and 5^′^ UTR for each gene. The average GC contents of CDS, introns, and 5^′^ UTR are 49.78 ± 7.04%, 42.54 ± 7.31%, and 65.08 ± 14.65%, respectively. Our results show that the GC content of 5^′^ UTR is significantly higher than that of CDS and introns (significance tested using 1-way analysis of variance). As shown in Table 1, the GC content of 5^′^ UTR is significantly and positively correlated with expression level, expression breadth, and maximum expression level, whereas that of CDS and introns, and GC_3_ are negatively correlated with expression level and expression breadth, and are not correlated with maximum expression level. To determine which variables contributed to the differences in gene expression pattern and how they may interact, we performed multiple linear regressions with the above variables, excluding those not contributing significantly through the use of the t-statistical analysis and with backward stepwise regression. The best combinations of variables were the GC content of 5^′^ UTR and GC_3_ (expression level, R^2^ = 0.0846, P < 0.0001; expression breadth, R^2^ = 0.1031, P < 0.0001). Stepwise selection model analysis indicated that the GC content of 5^′^ UTR is the major factor responsible for variance in gene expression pattern (expression level, R^2^ = 0.0542, P < 0.0001; expression breadth, R^2^ = 0.0684, P < 0.0001).

**Table 1:** Relationship between GC content and expression pattern

**GC content**	**Expression level**		**Expression breadth**		**Maximum expression**	
5^′^ UTR	r = 0.2328	P < 0.0001	r = 0.2614	P < 0.0001	r = 0.1155	P < 0.0001
CDS	r = − 0.1025	P < 0.0001	r = − 0.1105	P < 0.0001	r = − 0.0675	P = 0.0223
intron	r = − 0.0556	P < 0.0001	r = − 0.0723	P < 0.0001	r = − 0.0191	P = 0.2358
GC_3_	r = − 0.1378	P < 0.0001	r = − 0.1624	P < 0.0001	r = 0.0017	P = 0.6028

For each gene, the GC contents of CDS, introns and 5^′^ UTR were determined; GC_3_ were calculated by codonW 1.4.2.; chicken expression data were directly obtained from Rao et al. [15]; correlation analyses were performed by SAS Proprietary Software Release 8.1; significance tests were corrected for multiple testing by the Bonferroni step-down correction; multiple linear regression analysis indicated that GC content in genes could explain approximately 10% of the variation in gene expression.

GC content varies greatly between different genomic regions in chicken. This trend has been partly attributed to the recombination heterogeneity, since the mutagenic effects of recombination could result in a mutational bias toward G and C bases in regions with high recombination rates (GC-biased gene conversion). Recently, Rao et al. [15] compared the GC content of CDS and introns between regions with the highest recombination rate and those of null recombination in chicken and showed that the GC contents of CDS and introns in the top recombination regions are significantly greater than that of the regions without recombination. Therefore, a question that should be addressed is whether the pattern between GC content and expression is independent of the recombination process. In order to test this hypothesis, we estimated the recombination rate in 1 Mb windows across the chicken genome and computed the average gene expression level and expression breadth for each non-overlapping window. No significant correlation was detected between recombination rate and gene expression (expression level, r = 0.0061, P = 0.6073; expression breadth, r = 0.0166, P = 0.1453).

In most avian species, the karyotype contains both macro-chromosomes and micro-chromosomes unlike that of mammals. The chicken karyotype (2n = 78) is made up of 39 pairs of chromosomes, which are divided into five pairs of macro-chromosomes (GGA1–5), five pairs of intermediate chromosomes (GGA6–10) and 28 pairs of micro-chromosomes (GGA11–38) along with one pair of sex chromosomes (ZW female, ZZ male). Micro-chromosomes display a high recombination rate, high gene density and high GC content, which clearly distinguish them from macro-chromosomes [25]. Furthermore, it has been shown that the distribution of GC content in macro-chromosomes is narrow while, in micro-chromosomes, it is broader and shifts to higher values [3]. In order to test whether the impact of GC content on gene expression pattern differs or not among the three chicken chromosome groups, regression analyses between GC content and gene expression in each of the three groups were performed. Table 2 shows that the GC content of 5^′^ UTR was positively correlated with gene expression level, expression breadth, and maximum expression level for the three groups. The GC contents of CDS, introns, and GC_3_ were negatively and significantly correlated with expression level, expression breadth and maximum expression level for the intermediate-sized chromosomes and micro-chromosomes. For the macro-chromosomes, a similar trend was observed but with a slightly lower coefficient except for maximum expression level. Since the gene density (number of genes per Mb) of macro-chromosomes was significantly lower than that of micro-chromosomes [25], we assume that this explains the slight difference observed between macro-chromosomes and micro-chromosomes. However, gene density did not show any significant correlation with expression level and expression breadth (expression level, r = 0.0194, P = 0.4466; expression breadth, r = 0.0622, P = 0.3828).

**Table 2:** Relationship between GC content and expression pattern in three chromosome groups

**Chr group**	**GC content**	**Expression level**		**Expression breadth**		**Maximum expression**	
Macro (1–5)	5^′^ UTR	r = 0.2161	P < 0.0001	r = 0.2770	P < 0.0001	r = 0.1169	P < 0.0001
	CDS	r = − 0.0538	P = 0.0014	r = − 0.0623	P = 0.0003	r = − 0.0080	P = 0.7233
	intron	r = − 0.0455	P = 0.0208	r = − 0.0482	P = 0.0021	r = − 0.0442	P = 0.2358
	GC_3_	r = − 0.0596	P = 0.0342	r = − 0.0968	P < 0.0001	r = − 0.0223	P = 0.4972
Interm (6–10)	5^′^ UTR	r = 0.1978	P < 0.0001	r = 0.1400	P < 0.0001	r = 0.0624	P < 0.0001
	CDS	r = − 0.1689	P < 0.0001	r = − 0.1549	P < 0.0001	r = − 0.1377	P < 0.0001
	intron	r = − 0.1283	P < 0.0001	r = − 0.0923	P < 0.0001	r = − 0.0229	P = 0.0223
	GC_3_	r = − 0.1826	P < 0.0001	r = − 0.1786	P < 0.0001	r = − 0.1453	P < 0.0001
Micro (11–38)	5^′^ UTR	r = 0.1234	P < 0.0001	r = 0.2040	P < 0.0001	r = 0.1374	P < 0.0001
	CDS	r = − 0.1647	P < 0.0001	r = − 0.1850	P < 0.0001	r = − 0.1172	P < 0.0001
	intron	r = − 0.0961	P < 0.0001	r = − 0.1294	P < 0.0001	r = − 0.0562	P < 0.0001
	GC_3_	r = − 0.1719	P < 0.0001	r = − 0.1974	P < 0.0001	r = − 0.1171	P < 0.0001

Based on reference [23], the chicken autosomes were divided into five pairs of macro-chromosomes (Macro = GGA1–5), five pairs of intermediate chromosomes (Interm = GGA6–10) and 28 pairs of micro-chromosomes (Micro = GGA11–38); for each gene, the GC contents in CDS, introns, and 5^′^ UTR were determined; GC_3_ were calculated by codonW 1.4.2.; correlation analyses were performed by SAS Proprietary Software Release 8.1; significance tests were corrected for multiple testing by the Bonferroni step-down correction.

**Figure 1:**
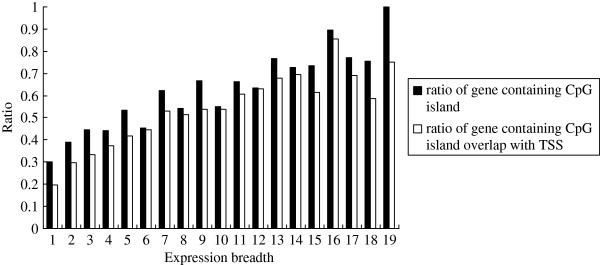
**Ratio of genes containing CpG islands according to different values of expression breadth.** CpG islands were identified using hgTables of the UCSC Genome Browser (http://genome.ucsc.edu/); the following search criteria were used: GC content ≥ 55%, Obs_CpG_/Exp_CpG_ ≥ 0.65, and length ≥ 500 bp; the ratio of genes containing CpG islands is significantly and positively correlated with expression breadth at a very high coefficient (r = 0.9342, P < 0.0001); the ratio of genes containing CpG islands overlapping with TSS is also highly correlated with expression breadth (r = 0.9114, P < 0.0001).

Based on the hgTables of the UCSC Genome Browser ( http://genome.ucsc.edu/), we analyzed CpG islands in the 5^′^ region of these genes, and more specifically in the promoter regions. Among the 8631 genes analyzed, 4393 contained at least one CpG island in the 2 kb region upstream of the first coding site. Among these 4393 genes, 3699 overlapped with a TSS. Of the 292 ubiquitously expressed genes (expression breadth ≥ 15), 232 contained CpG islands, and 30 genes had CpG islands that did not overlap with a TSS. The average size of the CpG islands was 934 bp. Of the 2743 tissue-specific genes (expression breadth ≤ 3), 1016 contained CpG islands, and 282 genes has CpG islands that did not overlap with a TSS. In this case, the average size of the CpG islands was 912 bp. Figure 1 shows the ratio of genes containing CpG islands and the ratio of genes containing CpG islands overlapping with a TSS within the 5^′^ region of genes according to different values of expression breadth. Regression analysis indicated that the ratio of genes containing CpG islands was significantly and positively correlated with expression breadth with a very high coefficient (r = 0.9342, P < 0.0001). The ratio of genes containing CpG islands overlapping with a TSS was also highly correlated with expression breadth (r = 0.9114, P < 0.0001).

## Discussion

In order to investigate whether the heterogeneous distribution of GC content along the genome has any significant impact on gene expression pattern in chicken, we carried out a genome scale analysis of the relationship between GC content and expression. Our data demonstrated that the GC content of the 5^′^ UTR was positively correlated with expression level, expression breadth, and maximum expression level, whereas, the GC contents of CDS, introns, and GC_3_ were negatively correlated with expression level and expression breadth. These significant trends are independent of recombination rate, chromosome size and gene density. Furthermore, multiple linear regression analysis indicated that the GC content could explain approximately 10% of the variation in gene expression. The best combinations of variables were GC content in 5^′^ UTR and GC_3_. Stepwise selection model analysis indicated that the GC content in 5^′^ UTR is the major factor responsible for the variance in gene expression pattern. Our study clearly demonstrated that the nucleotide composition of a gene is significantly associated with gene expression pattern and could be an important regulation factor in chicken.

The positive trend between GC content in 5^′^ UTR and expression could be due to the C → T mutation bias of that has been selectively hampered by depletion of methylation of cytosine in CpG dinucleotides from the CpG islands in the 5^′^ regions of genes. Indeed, it is well known that, in vertebrates, most CpG dinucleotides are methylated at the carbon 5-position of the cytosine residue except for the CpG dinucleotides in CpG islands, and a 5-methylcytosine is more likely to be deaminated to produce a thymine (C → T mutation bias) [26,27]. Recently, Li et al. [28] constructed a genome-wide chicken DNA methylation map. They found that DNA methylation is enriched in the gene body regions and the repetitive sequences, and depleted in transcription start and transcription termination sites. In our study, we also found that the GC content of 5^′^ UTR is significantly higher than that of CDS and introns (significance tested using 1-way analysis of variance). Similar to previous studies [10,25,28,29], our data indicate that CpG islands typically occur at the TSS of genes, in particular for ubiquitously expressed genes. Furthermore, regression analysis showed that the ratio of genes containing CpG islands overlapping a TSS was significantly and positively associated with expression breadth at a very high coefficient. This implies that a gene containing CpG islands overlapping a TSS tends to be expressed in many tissues in chicken. Since expression level and expression breadth are highly correlated in this dataset (r = 0.8348, P < 0.001), the same biased mutational process discussed above could explain the significant trend between GC content in 5^′^ UTR and gene expression level in chicken. Since 5^′^ UTR regions are known to play crucial roles in post-transcriptional regulation of gene expression [30], the nucleotide composition of these regions may evolve partly in response to selective pressures depending on their function.

There is no clear explanation for the negative correlation between gene expression and GC content in CDS, introns and GC_3_. A weak negative correlation between GC content in CDS and expression breadth has also been reported in man by Gonçalves et al. [10]. They compared the structure and expression of genes with or without known retropseudogenes, and found that genes with retropseudogenes show significantly poorer GC levels than genes without known retropseudogenes, suggesting that GC-poor mRNA have a higher efficiency of reverse-transcription through LINE reverse-transcriptase compared with GC-rich mRNA. Since retrotranscribed genes are predominantly ubiquitously expressed genes involved in metabolism or in protein and RNA synthesis in human, the negative correlation between GC level and expression breadth can be partially explained. However, this is not the case in the chicken genome. Although the chicken genome hosts its own LINE-like elements, the reverse transcriptase encoded by these elements is unlikely to copy polyadenylated mRNA since the density of LINEs and the activity of transposable elements are very low. Recently, Rao et al. [15] carried out a systematic examination of the codon usage in chicken. They found that codon bias is negatively correlated with GC_3_, GC content in CDS, and GC content in intronic sequences with a high coefficient. Combined with the results of this study, we conclude that a gene with a lower GC content in CDS, introns, or GC_3_ tends to be highly and broadly expressed among tissues with stronger codon bias in chicken. This negative trend may be explained partially by selection of these genes to translate more efficiently and accurately in the chicken genome.

## Conclusions

GC content in genes is significantly associated with gene expression pattern and could be one of the important regulation factors in the chicken genome.

## Competing interests

The authors declare that they have no competing interests.

## Authors’ contributions

YSR, XQZ and XWC conceived and designed the experiments. ZFW and QHN analyzed the data. XWC and ZFW collected the expression data. YSR and XQZ wrote the paper. All authors read and approved the final manuscript.
